# Genetic variances, heritabilities and maternal effects on body weight, breast meat yield, meat quality traits and the shape of the growth curve in turkey birds

**DOI:** 10.1186/1471-2156-12-14

**Published:** 2011-01-25

**Authors:** Muhammad L Aslam, John WM Bastiaansen, Richard PMA Crooijmans, Bart J Ducro, Addie Vereijken, Martien AM Groenen

**Affiliations:** 1 Animal Breeding and Genomics Centre, Wageningen University, 6709PG, Wageningen, the Netherlands; 2 Hendrix Genetics, Research & Technology Centre, 5830 AC, Boxmeer, the Netherlands

## Abstract

**Background:**

Turkey is an important agricultural species and is largely used as a meat bird. In 2004, turkey represented 6.5% of the world poultry meat production. The world-wide turkey population has rapidly grown due to increased commercial farming. Due to the high demand for turkey meat from both consumers and industry global turkey stocks increased from 100 million in 1970 to over 276 million in 2004. This rapidly increasing importance of turkeys was a reason to design this study for the estimation of genetic parameters that control body weight, body composition, meat quality traits and parameters that shape the growth curve in turkey birds.

**Results:**

The average heritability estimate for body weight traits was 0.38, except for early weights that were strongly affected by maternal effects. This study showed that body weight traits, upper asymptote (a growth curve trait), percent breast meat and redness of meat had high heritability whereas heritabilities of breast length, breast width, percent drip loss, ultimate pH, lightness and yellowness of meat were medium to low. We found high positive genetic and phenotypic correlations between body weight, upper asymptote, most breast meat yield traits and percent drip loss but percent drip loss was found strongly negatively correlated with ultimate pH. Percent breast meat, however, showed genetic correlations close to zero with body weight traits and upper asymptote.

**Conclusion:**

The results of this analysis and the growth curve from the studied population of turkey birds suggest that the turkey birds could be selected for breeding between 60 and 80 days of age in order to improve overall production and the production of desirable cuts of meat. The continuous selection of birds within this age range could promote high growth rates but specific attention to meat quality would be needed to avoid a negative impact on the quality of meat.

## Background

Turkey is largely used as a meat bird. In 2004, turkey represented 6.5% of the world poultry meat production [1]. The world-wide turkey population has rapidly grown due to increased commercial farming. Global turkey stocks increased from 100 million in 1970 to over 276 million in 2004. Over the same time period, the production volume increased from 1.2 to 5.1 million [1]. Due to the high demand for turkey meat from both consumers and industry, the breeding objective is to produce rapidly growing birds with a high market body weight (BW) and a desirable body conformation in order to maximize production efficiency and optimize production of preferred body cuts; e.g., breast muscle [2]. These objectives can be achieved by selective breeding of birds for high body weights, with much emphasis on breast muscle yield, while considering the efficiency of production over the growth curve. Knowledge of the growth curve will be useful when defining ages and weights at which to select birds as well as for the design of management procedures.

Breeding programs for meat type birds are commonly selecting for BW, and body composition traits (breast yield, etc.) while minimizing production costs. Recently breeders have started to measure meat quality (drip loss, pH, etc.) as well as survival traits, at least in research projects [3,4]. Selection was found successful to improve growth and body composition traits while these traits did not show any negative association with the excessive drip loss in chicken [3,5]. Drip loss was found correlated with pH of meat and differences in pH significantly affect the storage and the processing quality of the meat [5,6]. Meat with low pH is characterized by a low water-holding capacity and poor technological quality and is therefore referred to as pale, soft, and exudative (PSE) meat [6,7]. Meat with high pH, known as dry, firm, and dark (DFD) meat, is characterized by a poor storage quality which is the result of a faster rate of off-odor production and an accelerated microbiological growth [8].

BW traits were found to be influenced by not only genetics but also common or maternal environmental effects [9]. Nestor et al. [10] reported that the un-weighted averages of published narrow sense heritability (***h^2^***) estimates of BW in selected populations of turkey birds were 0.40, 0.42, 0.43, and 0.36 for birds in the age groups 0 to 8, 9 to 16, 17 to 24, and over 24 wk, respectively [10]. Other studies also found high heritabilities for BW at various ages, ranging from 0.28 to 0.48 [11-13]. Strong positive genetic correlations were found between the 16-wk BW and BW at other ages (8, 20, and 24 wk of age). Negative correlations were found between BW and reproduction traits [13]. Toelle et al. [14] found that the genetic correlation between BW of the two sexes at 16 wk of age was close to unity.

Many reports exist that show estimation of growth curves; an understanding of growth curves is important for the efficient production of animals [15]. Growth curve parameters were estimated for turkeys by Sengul and Kuraz [16] with four different non-linear models (Gompertz, Logistic, Morgan-Mercer-Flodin [MMF], and Richards) and very good fits were found with Gompertz, Logistic and Richards models. Mignon-Grasteau et al. [17] estimated growth curve parameters with the Gompertz function in chickens. High heritabilities were found for these growth curve parameters [17]. It was established that the growth curve varies among individuals; thus, growth might be enhanced by selection on the basis of growth curve parameters [18].

In this study, we estimated genetic parameters for different growth (BW and growth curve traits) and meat quality traits in turkeys as well as the genetic and phenotypic correlations between these traits. To our knowledge, this is the first study to evaluate genetic parameters for meat quality in turkey and to estimate correlations of turkey meat quality with growth traits and meat yield traits.

## Methods

### Animals

The study population was based on two genetically different commercial turkey lines referred to as line A and line B. Line A was selected for rapid growth and line B was selected for a high reproduction rate. Males from line A were crossed to females from line B to produce F1 offspring. From the F1 generation, 25 males and 34 females were randomly selected and mated to produce 1,716 F2 offspring. The number of F2 offspring in a full-sib group ranged from 16 to 120 with an average of 63 offspring per group. Each F1 female was mated once; therefore the pedigree included no maternal half-sibs. F2 individuals had pedigree information for 9 generations and phenotypes were recorded only on F2 individuals. The pedigree consisted of 2,186 individuals; the F2 individuals were from 14 different hatch dates between 21-05-2000 and 04-11-2001. The package pedigree, in R statistical software [19], was used to check the pedigree file for potential errors.

### Feeding Schedule

Turkey birds were fed according to the feed schedule and nutrient guidelines of Hybrid (A Hendrix Genetics Company). Feed changed in energy (ME/Kg), crude protein percent (CP) and other essential nutrients level with the age of a bird. Energy level of feed was raised while CP level was lowered with increasing age of birds. In the 1^st ^week, feed was supplied with a CP level of 27.5% and an energy level of 2850 ME/Kg while in the 17^th ^week of age CP level had been lowered to 17% and energy level had been raised to 3520 ME/Kg.

### Housing Conditions

Turkey birds were raised in unisex groups of around 500 to 525 poults/group. The bedding material was comprised of wood shavings for the entire rearing period, and in the first week of age, brooder rings were used. The birds were kept in closed barns with concrete floors and controlled lighting and ventilation systems. The same duration of light (12 hr/day) was provided to both male and female birds during the first 15 weeks. After 15 weeks, light was provided for 14 hr/day and 16 hr/day to male and female birds, respectively. The environmental temperature was maintained at a relatively high level of 22.8 to 27.8°C during the first week, after which it was decreased gradually with the age of the birds. After 12 weeks, the temperature was kept constant at 13.9 to 16.1°C. In the first 6 weeks, birds were provided floor space of 0.074m^2^/bird. After 6 weeks, the floor space was increased to 0.167 m^2^/female and 0.185 m^2^/male up to 15 weeks; the final floor space of 0.209 m^2^/female and 0.269 m^2^/male was provided during 16 to 20 weeks of age.

### Traits

Phenotypic data were recorded as part of a commercial breeding program. BW and carcass related traits were recorded for 1,716 (692 females and 1,024 males) individuals of the F2 generation. Body weights were recorded at 1, 17, 40, 60, 80, and 120 days (BW01, BW17, BW40, BW60, BW80, and BW120, respectively). The breast meat yield traits breast length (BrL) and breast width (BrW) were measured with a caliper in live birds just before slaughter at 20 weeks of age. BrW was measured at the widest point of the breast while BrL was measured at the symmetry line of the breast. The percent breast meat (PBM) and percent drip loss (PDL) were recorded at 20 weeks of age after the birds were slaughtered. PDL was measured in breast meat samples of 30 to 50 g. After measuring initial weight, samples were packed and hung for five days at a temperature of 4°C. After a storage period of five days, the samples were weighed again for the final weights. The PDL was recorded as a percentage of initial weight [20].

The ultimate pH (pHu) of the *Pectoralis major *muscle of a skinless breast fillet was measured at 24 h post-slaughter with a piercing electrode (Cole Parmer L-05992-22, Chicago, Illinois). Breast meat color was measured at 24 h post-slaughter using a portable Minolta Chroma Meter (Model CR-200; Ramsey, NJ) with the CIE L*a*b* system, where L* represents lightness, a* redness and b* yellowness. Higher L*, a* and b* values correspond to paler, redder and more yellow meat, respectively. The Minolta Chroma Meter was calibrated according to the CIELAB color system. The pH and color were measured in the same area of the breast, on the thickest position of the lobe.

### Growth Curve

Growth curve parameters were estimated with a logistic growth function (SSlogis) in R statistical software [21]. Only individuals that had measurements for BW01 and BW120 and at least 2 additional BW measurements were included for the estimation of growth curve parameters. With these restrictions 867 out of the total 1,716 birds were included. Population parameter values of the logistic growth curve were estimated for the male and female populations separately as well as sex average parameter values. Growth curves were plotted for every individual in a population using their estimated parameter values. Separate logistic growth curves were also plotted for the male and female populations as well as the complete population with the estimated parameter values. To estimate the parameters of the logistic growth curve, the following equation was fitted to the data:

$$W_{t}=\frac{As_{wt}}{1+e^{t}{{}^{mid}}^{-t/scale}}$$

where W(t) is weight at time t (days), **As_wt _**is the asymptotic weight (Kg), **t_mid _**is the inflection point at which 50% of the asymptotic weight is achieved (days), and **scale **is a constant that is proportional to the overall growth rate [22,23].

### Genetic Analyses

Descriptive statistics were obtained from a generalized linear model (PROC GLM [24]). The correction of data and removal of outlier values (>3SD) and the test for the normality of the distribution of traits was performed with method PROC UNIVARIATE [24]. Only PBM and PDL displayed outlier values (>3SD) and those animals were removed from the analysis. Fixed effects of sex and hatch date were tested for significance of their effect on each trait with PROC GLM [24]. Effects that were found significant (*P *< 0.05) were included in the model for the estimation of genetic parameters.

Heritabilities for all the traits under study were estimated with an animal model in ASREML statistical software [25] using univariate analyses. Bivariate analyses for all possible combinations of traits were applied to estimate genetic and phenotypic correlations. Estimates obtained in univariate analysis were used as starting values in bivariate analyses. In the ASREML program, the maximum number of iterations was set to 20; for the most part, convergence criteria were met in less than 10 iterations and always before 20 iterations. An additional 10 iterations after convergence did not change results. Convergence was presumed when the log-likelihood changed less than 0.002 between iterations and the individual variance parameter estimate changed less than 1% [25].

In addition to the genetic analyses mentioned above, the genetic correlations between BW of males and BW of females at the same age (e.g. BW01 M and BW01F) were also estimated for each BW trait using a bivariate analysis to test if male and female growth should be regarded separate traits.

A random common environment effect of the dam was included in the model for all the traits, except for meat quality traits (PDL, pHu, L*, a* and b*). A likelihood ratio test (**LR-test**) was used to check the significance of the full model (with a random common environment of dam) compared to the reduced model (without a random common environment of dam) based on the following equation:

$$Y_{ijkl}=\mu +S_{i}+H_{j}+A_{k}+C_{l}+E_{ijkl}$$

Where Y_ijkl _is the performance of individual *k*, μ is overall mean, S_i _is the fixed effect of sex *i*, H_j _is the fixed effect of the week of hatch *j *(*j *= 1, 2...14), A_k _is the random direct genetic effect of individual *k *with $a\sim N\left(0,A\sigma_{a}^{2}\right)$, C_l _is the random common environment effect of the *l*-th dam with $c\sim N\left(0,I\sigma_{c}^{2}\right)$, and E_ijkl _is the random residual effect.

### Ethical approval for the use of animals in this study

Although animals were used in this work, no experiments were performed on them. Data was recorded as a part of the routine work at a breeding company (Hendrix Genetics). No approval from the ethics committee was necessary.

## Results

### Descriptive Analysis

A descriptive analysis of all the traits studied was summarized in Table 1. The effect of sex was significant (P < 0.05) for all the traits except for the weight of 1 day old chicks (BW01) and the redness of meat (a*). The mean values for all the traits studied were higher for males than females. The effect of hatch date was also significant for all the traits.

**Table 1:** Descriptive statistics, including the estimates for the significant fixed effects (Sex and Hatch).

Traits(units)	N	Minimum	Maximum	LS Mean	RSD	**Sex**^**1**^	**Hatch**^**2**^
**BW01(Kg)**	1416	0.04	0.07	0.06	0.00	0.00	0.02***
**BW17(Kg)**	1281	0.08	0.60	0.35	0.05	0.21***	0.11***
**BW40(Kg)**	1226	0.52	2.32	1.40	0.17	0.66***	0.37***
**BW60(Kg)**	1103	1.50	4.96	3.18	0.37	1.27***	0.65***
**BW80(Kg)**	1009	3.04	8.50	5.57	0.59	2.22***	1.64***
**BW120(Kg)**	878	4.54	15.90	10.49	1.01	5.00***	1.48***
**PBM (%)**	919	9.10	13.40	11.19	0.71	-0.20***	1.17***
**BrL(mm)**	1198	149.00	249.00	196.35	14.08	49.44***	23.19***
**BrW(mm)**	1198	107.00	181.50	135.93	7.91	26.70***	21.80***
**PDL(%)**	1028	2.21	14.10	5.11	1.14	0.94***	1.36***
**pHu**	1055	5.22	6.08	5.73	0.09	0.03***	0.49***
L*	1083	40.30	53.60	45.94	1.72	1.03***	2.65***
a*	1083	1.30	9.20	5.25	0.97	0.06	1.06***
b*	1083	0.00	5.60	2.25	0.77	0.51***	0.81***
**As_wt _(Kg)**	867	4.6	20.23	12.39	1.32	6.47***	2.82***
**t_mid_(day)**	867	59.86	112.24	82.82	3.58	6.07***	13.20***
**scale(day)**	867	12.66	29.15	20.61	1.21	1.86***	5.78***

N = Number of records; minimum = minimum values; maximum = maximum values; LS Mean = least square mean; RSD = residual standard deviation; BW01, BW17, BW40, BW60, BW80, and BW120 are the BW at days 1,17, 40, 60, 80, and 120 of age, respectively; PBM = percentage breast meat at 20 week of age; BrL = breast length at 20 week of age; BrW = breast width at 20 wk of age; PDL = percent drip loss at 20 week of age; pHu = ultimate pH at 20 wk of age; L* = lightness at 20 wk of age; a* = redness at 20 wk of age; b* = yellowness at 20 wk of age; As_wt _= upper asymptotic weight (estimated growth curve parameter); t_mid _= inflection point at 50% asymptote (estimated growth curve parameter); scale = constant that is proportional to the overall growth rate (estimated growth curve parameter). ^1 ^= The difference between sexes in the Least square means (LS Means) of the traits. ^2 ^= The difference between the maximum and minimum LS Means of the traits with respect to the week of hatch. *P ≤ 0.05, **P ≤ 0.005, ***P ≤ 0.0005

### Growth Curve

**Figure 1:**
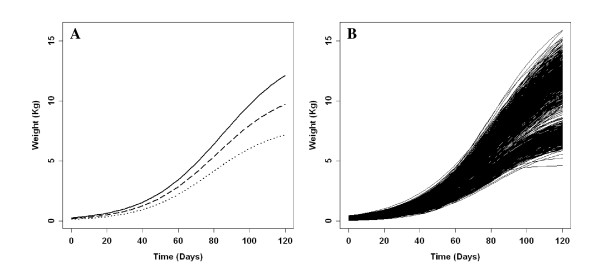
**Logistic growth curves depicting the change in growth rate of the turkey population through time**. A = Growth curves representing average growth rates in males (solid line), females (dotted line), and sex average (dashed line); B = Growth curves of all the individuals in the population.

The average parameter values estimated from the logistic growth curve are given in Table 2. The logistic growth curves were estimated and plotted from actual measurements of BW throughout the growth period; in this case, BW01, BW17, BW40, BW60, BW80, and BW120 (Figure 1). The male and female populations showed a difference in growth rate that was apparent in the estimates of the growth curve parameters and could also be observed in Figure 1B which shows an apparent split into 2 groups of the individual animal growth curves.

**Table 2:** Estimates of logistic growth curve parameters for males, females, and sex average parameter values.

	**As**_**wt**_**(Kg)**	**t**_**mid**_**(day)**	scale(day)
**Male**	14.44	84.87	21.39
**Female**	7.88	78.28	19.22
**Sex average**	11.16	81.58	20.31

As_wt _= upper asymptote (estimated growth curve parameter); t_mid _= inflection point at 50% asymptote (estimated growth curve parameter); scale = constant that is proportional to the overall growth rate (estimated growth curve parameter).

### Heritability Estimates

Body weight traits BW40, BW60, BW80, and BW120 were found to be highly heritable, with heritability estimates (***h^2^***) of 0.32, 0.39, 0.42, and 0.40, respectively (Table 3). The BW at 1 and 17 days (BW01 and BW17) were found to have low heritability, with estimates of 0.0 and 0.12 respectively. The proportion of variance explained by common (maternal) environment was 0.43 at BW01. This proportion reduced rapidly to 0.11 at BW17 and became negligible after BW60.

**Table 3:** Estimates of heritability, standard deviations and common environment variance ratios for different traits.

Trait	***σ ***_***a***_	***σ ***_***c***_	***h***^***2 ***^**(S.E)**	***C***^***2 ***^**(S.E)**
**BW01(Kg)^1^**	0.0045	2.99	0.00(0.00)	0.43(0.06)
**BW17(Kg)^1^**	18.25	17.11	0.12(0.20)	0.11(0.08)
**BW40(Kg)**	99.60	36.36	0.32(0.22)	0.04(0.07)
**BW60(Kg)**	241.17	41.13	0.39(0.26)	0.01(0.08)
**BW80(Kg)**	396.29	0.06	0.42(0.12)	1.09E-08(0.00)
**BW120(Kg)**	652.68	0.04	0.40(0.12)	1.86E-09(0.00)
**PBM(%)**	0.39	7.11E-10	0.30(0.10)	1.36E-09(0.00)
**BrL(mm)**	5.51	4.80E-06	0.15(0.06)	2.37E-08(0.00)
**BrW(mm)**	3.30	1.12E-07	0.17(0.07)	1.73E-09(0.00)
**PDL(%)**	0.40	NI	0.12(0.06)	NI
**pHu**	0.03	NI	0.09(0.04)	NI
L*	0.90	NI	0.27(0.09)	NI
a*	0.54	NI	0.30(0.09)	NI
b*	0.30	NI	0.15(0.05)	NI
**As_wt_(Kg)**	737.68	0.07	0.30(0.10)	2.67E-09(0.00)
**t_mid_(day)**	0.80	0.00021	0.05(0.04)	3.44E-09(0.00)
**scale(day)**	0.41	0.00037	0.11(0.06)	8.99E-08(0.00)

*σ _a _*= Additive genetic standard deviation; *σ _c _*= common environment standard deviation; *h*^2 ^(S.E) = heritability estimates with standard errors (S.E); *c*^2 ^(S.E) = common environment variance ratio with standard errors (S.E); BW01, BW17, BW40, BW60, BW80, and BW120 are the BW at days 1,17, 40, 60, 80, and 120 of age; PBM = percentage breast meat at 20 wk of age; BrL = breast length at 20 wk of age; BrW = breast width at 20 wk of age; PDL = percent drip loss at 20 wk of age; pHu = ultimate pH at 20 wk of age; L* = lightness at 20 wk of age; a* = redness at 20 wk of age; b* = yellowness at 20 wk of age; As_wt _= upper asymptote (estimated growth curve parameter); t_mid _= inflection point at 50% asymptote (estimated growth curve parameter); scale = constant that is proportional to the overall growth rate (estimated growth curve parameter). NI = Not Included (common environment) in the analysis for the trait. **^1 ^**= Full model with common environment effect was found significantly different from the reduced model for these traits; *P *< 0.05.

The heritability estimates for breast meat yield traits PBM, BrL, and BrW were in the moderate to high range, with estimates of 0.30, 0.15, and 0.17, respectively.

The meat quality traits PDL, pHu, L*, a* and b* showed low to high estimates of heritability. PDL and pHu showed low heritabilities of 0.12 and 0.09 respectively while the other quality traits L*, a* and b* showed moderate to high heritabilities with estimates at 0.27, 0.30 and 0.15 respectively.

The growth curve trait As_wt _showed a high heritability estimate of 0.30, and the remaining two growth curve traits, t_mid _and scale, showed lower heritabilities at 0.05 and 0.11, respectively.

### Genetic and Phenotypic Correlations

Genetic and phenotypic correlations were estimated between all the BW traits (BW17, BW40, BW60, BW80, and BW120), except BW01, which showed zero heritability. We found high positive genetic correlations among all the BW traits ranging from 0.86 to 0.98 (Additional file 1). Genetic correlations decreased as the time between BW measurements increased, except for correlations with BW120. At this point, the birds were well past the inflection point (Figure 1) and were close to their final adult BW. Phenotypic correlations among all the BW traits were also found to be high and positive. Genetic correlations between BW of males and BW of females at the same age were found to be high in the range of 0.87 - 0.99 for all BW traits. BW measures were therefore treated as one trait in subsequent analyses.

Positive genetic correlations were also found among the breast meat yield traits, BrL, BrW, and As_wt _which ranged from 0.68 to 0.90. These traits also showed high positive phenotypic correlations. All BW traits and As_wt _showed genetic correlations close to zero with PBM, albeit with large standard error of estimates. Positive phenotypic correlations of PBM with BW traits and As_wt _ranged from 0.21 to 0.32. The As_wt, _BrL, and BW traits showed positive genetic and phenotypic correlations with PDL, lightness and yellowness (L* and b*). The traits pHu and a* showed negative genetic correlation with As_wt, _BrL, and BW traits but these results had a high standard error of estimates. Phenotypic correlation of pHu and a* with As_wt, _BrL, and BW traits was close to zero. The ultimate pH had negative genetic and phenotypic correlations with PDL with the genetic correlation estimated close to minus one (Additional file 1). PDL showed positive genetic and phenotypic correlations with L*, a* and b* while L* had negative genetic and phenotypic correlations with a* and positive genetic and phenotypic correlations with b*. The genetic and phenotypic correlations between a* and b* were close to zero. The growth curve parameter t_mid _showed a highly negative genetic correlation with PDL, and phenotypic correlations that were either negative or close to zero with all other traits except for the other two growth curve traits As_wt _and scale. Genetic and phenotypic correlations between the PBM and PDL were close to zero.

Positive genetic and phenotypic correlations were found among PBM, BrL, and BrW. BrL showed positive genetic and phenotypic correlations with PDL. In contrast, BrW showed a negative genetic correlation and a positive phenotypic correlation with PDL. All the correlations of BrL and BrW with PDL were however close to zero (Additional file 1).

The analysis of growth curve traits showed that As_wt _had negative genetic correlations, but positive phenotypic correlations, with t_mid _and scale. Positive genetic and phenotypic correlations were observed between t_mid _and scale.

## Discussion

The aim of this study was to estimate heritabilities and determine genetic and phenotypic correlations for BW, breast meat yield, and meat quality traits in turkeys. We also aimed to estimate the growth curve and the heritabilities of its parameters. The phenotypes used in this study were measured on an F2 cross between 2 turkey lines with a different genetic background and selected for different traits. The variances obtained are relevant to the F2 cross and cannot be directly applied to existing breeding stock. The estimates do provide a useful benchmark for breeders interested in the potential for correlated responses in meat quality from selection on growth and yield and for breeders who contemplate the estimation of heritabilities in their breeding lines and/or adding these traits to their breeding objectives.

In the present study, body weight was considered to be a single trait across both sexes, with sex used as a fixed effect in the analyses. This was in contrast with other studies, where parameters were estimated separately for males and females [13,26,27]. Parameters were not estimated separately in our analyses because those estimates would have been based on a subset of our relatively small population. Joint analysis of males and females seems warranted because genetic correlations between BW of males and BW of females at the same age were found to be high. In addition to sex, hatch date was included as a fixed effect in the analyses because it was found to play a significant role in BW and other traits in the study [28,29].

In the present study, univariate models were used for the estimation of heritability and bivariate models for the estimation of genetic and phenotypic correlations [30]. Multivariate analyses were performed for small groups of related traits and results were not different from those obtained from univariate and bivariate models. Combining traits did not always result in convergence of the REML estimation. A common environmental variance (***c^2^***) was found significant for some traits (BW01 & BW17) and not for others which further complicated the estimation from multivariate models.

We found heritability estimates for BW traits in the expected range, except for BW01 and BW17, which is attributed to the strong common environment effect at those early ages. Results are in range with previously reported heritability estimates. BW traits at various ages were reported to have an average heritability of 0.41 in a review of eighteen reports by Arthur and Abplanalp [11]. Similar results were also reported by Buss [31], who observed heritability in the range of 0.23 to 0.71 for BW traits at different ages.

The common environment effect had a large impact on the estimates of heritability for BW traits, especially at early ages. Neglecting the common environment effect would have resulted in an overestimation of heritabilities at early ages. For comparison, we estimated heritabilities without including the common environmental effect (results not shown), and found that the estimated heritability of body weight was increased at all ages, but especially for BW01 and BW17. Similar conclusions were reached by others regarding the effect of common environment on the estimation of heritability [12,32-34]. In our study, *c^2 ^*was found to decrease with increases in age and it was close to zero at later ages. The direct genetic component was found to increase with age which could be attributed to the initiation of expression of the animal's own genetics.

In the present study, the BW of day old turkey chicks had a heritability close to zero. Tullett and Burton [35] found in a study on broilers that 97% of the variation in chick weight at hatching was due to two factors: fresh egg weight and weight loss during incubation. Moreover, North [36] found that egg weight represented 70% of the chick weight. Taken together, these results suggest that day old BW was not heritable, but egg weight or egg size was heritable.

Our heritability estimates of the other production traits, including PBM, BrL, and BrW, were also consistent with reports from other groups. Our heritability estimate for PBM was 0.30, similar to values found by Le Bihan-Duval et al. [2] in chickens. The comparison is made to chicken because it is the closest related species to the turkey for which values are available. Our heritability estimates for breast length and breast width were low and quite close to each other. These results were in agreement with the work of Adeyinka et al. [28] on chickens. Our heritability estimate for PDL at 0.12 was the first reported for turkey meat, and somewhat inconsistent with the heritability of 0.26 found in chickens by Le Bihan-Duval et al. [2]. Besides the estimate being made in different species there were also differences in the measurement of traits with Le Bihan-Duval et al. [2] measuring PDL from the whole breast muscle while a smaller breast meat sample was used in our study.

The heritabilities in the present study for pHu, a* and b* at 0.09, 0.30 and 0.15 were found roughly in agreement with the results of Le Bihan-Duval et al. [37] in turkeys, while our estimate of heritability for L*, 0.27, was somewhat higher that the value of 0.12 obtained Le Bihan-Duval et al. [37]. A possible explanation can be sought in the different fixed effects included in the models by these two studies which in turkey may have explained a bigger part of the residual variance for this particular trait L*.

Sengul and Kuraz [16] concluded that Gompertz, Logistic, and Richards models all performed well for describing growth in turkeys. The logistic and Gompertz models have fixed growth forms with points of inflection at about 50 and 37% of the asymptote, respectively [22]. These parameter models are special cases of the more flexible Richards model, which has a variable point of inflection specified by the shape parameter [38]. The growth models (Logistic, Gompertz and Richards) also differ slightly from each other in the interpretation of other parameters [39]. Here, we choose to use the logistic growth model for the analyses of growth. The As_wt _(upper asymptote) had high heritability, consistent with that found by Mignon-Grasteau et al. [17] who used the Gompertz model in chickens. We found low heritability estimates for t_mid _and scale which was not in agreement with the results reported by Grossman and Bohren [40] in chicken but the heritability estimate for t_mid _from our study was in agreement with the results from Le Rouzic et al. [41] in chicken who used a Gompertz growth model. Inconsistency in the results of the present study and the study by Grossman and Bohren [40] for t_mid _and scale could be due to the difference in species, differences between methods for the estimation of genetic parameters (based on correlation among full-sibs in Grossman and Bohren [40]) or because of the high margin of error reported in the study by Grossman and Bohren [40]. The differences we observed between the estimates of growth curve parameters for males and females were similar to differences observed by Sengul and Kuraz [16] in white turkeys and by Barbato and Younken [42] in chickens.

In the present study, the genetic correlations among all the BW traits ranged from 0.86 to 0.99. Genetic correlations were higher for measurements taken close together in age and declined somewhat as the measurement were taken farther apart in age. Similar results on genetic correlations among multiple BW traits were reported by Kranis et al. and Chapuis et al. [12,26], who applied various mixed models and performed multivariate analyses. We found high genetic and phenotypic correlations among all the BW traits and the As_wt_; the correlations generally increased as the age of the birds increased. Genetic and phenotypic correlations between the BW120 and As_wt _were both found close to 1, reflecting the similarity of the upper asymptote and BW at the later ages. The parameters t_mid _and scale showed a strong positive genetic and phenotypic correlation while both have negative genetic and positive phenotypic correlations with As_wt_. The negative genetic correlation between As_wt _and t_mid _is considered favorable since individuals with high As_wt _will take less time to reach t_mid _making that individuals with high asymptotic weight can be identified earlier. Similarly, positive genetic correlation between t_mid _and scale is also considered favorable and logical because for birds that take less time to reach 50% of the asymptotic weight we will automatically see shrinkage in the scale. A smaller value for scale also means asymptotic weight will be approached earlier. In other studies a negative genetic correlation was also observed between As_wt _and exponential rate of decay of the specific growth rate (k) by Mignon-Grasteau et al. [17] and between As_wt _and scaling parameter by Narinic et al. [43] who applied the Gompertz model in their work on chickens and quails respectively.

In our study, pHu showed highly negative genetic correlations with PDL, a* and b* whereas correlation with L* was moderately negative. These negative genetic correlations of pHu were in agreement with the previous work of Le Bihan Duval et al. on turkey and chicken [2,5,37]. The increase in positive genetic correlation of PDL with BW traits at later ages could be due to the increase in glycogen contents of breast muscles with age, which also had a strong negative genetic correlation with pHu [2]. The negative genetic correlation of pHu with L* and b* would explain off color meat (PSE) with low pHu and high drip loss and vice versa which was in agreement with the results from previous studies [6,7].

In our study, both the PDL and PBM were recorded in percentages, and the genetic and phenotypic correlations between these traits were close to zero. We found that PBM had positive genetic and phenotypic correlations with BrL and BrW. The high genetic and phenotypic correlation between BrL and BW traits was also observed by Adeyinka et al. [28] in chickens. The positive genetic and phenotypic correlation of PBM with BrL and BrW will be useful in selection for increased PBM which is an important trait but can only be recorded after the animal is killed.

## Conclusion

The results of this analysis, in particular the correlations between weights as well as the growth curve traits (Additional file 1), suggest that the turkey birds could be selected for breeding at earlier time points, between 60 and 80 days of age, in order to improve overall production and the yield of desirable cuts of meat at slaughter age. The selection of birds within this age range for high BW would also increase growth rates. Attention would need to be given to meat quality traits, drip loss and pHu which had low heritabilities but quality of meat would still be expected to deteriorate from selection on early body weight.

## Authors' contributions

MG, RPMAC and AV designed the study, MLA, JB and BD analyzed the data. MLA and JB wrote the manuscript and all other authors gave suggestions and comments for the improvement. All authors read and approved the final manuscript.

## Supplementary Material

Additional file 1 **Estimated genetic parameters (heritabilities and correlations with standard errors) for different traits in turkey birds**. Heritabilities (diagonal), genetic correlations (above the diagonal) and phenotypic correlations (below the diagonal) are presented with standard errors (in parenthesis) for the different traits. BW01, BW17, BW40, BW60, BW80, and BW120 are the BW at days 1,17, 40, 60, 80, and 120 of age; PBM = percentage breast meat at 20 wk of age; BrL = breast length at 20 wk of age; BrW = breast width at 20 wk of age; PDL = percent drip loss at 20 wk of age; pHu = ultimate pH at 20 wk of age; L* = lightness at 20 wk of age; a* = redness at 20 wk of age; b* = yellowness at 20 wk of age; As_wt _= upper asymptote (estimated growth curve parameter); t_mid _= inflection point at 50% asymptote (estimated growth curve parameter); scale = constant that is proportional to the overall growth rate (estimated growth curve parameter). Click here for file
