# Supplementary material for: Genetic variances, heritabilities and maternal effects on body weight, breast meat yield, meat quality traits and the shape of the growth curve in turkey birds
Source: BMC Genet. 2011 Jan 25;12:14. doi: 10.1186/1471-2156-12-14 (PMC3039623; doi:10.1186/1471-2156-12-14)
Supplement: Additional file 1 — Estimated genetic parameters (heritabilities and correlations with standard errors) for different traits in turkey birds. Heritabilities (diagonal), genetic correlations (above the diagonal) and phenotypic correlations (below the diagonal) are presented with standard errors (in parenthesis) for the different traits. BW01, BW17, BW40, BW60, BW80, and BW120 are the BW at days 1,17, 40, 60, 80, and 120 of age; PBM = percentage breast meat at 20 wk of age; BrL = breast length at 20 wk of age; BrW = breast width at 20 wk of age; PDL = percent drip loss at 20 wk of age; pHu = ultimate pH at 20 wk of age; L* = lightness at 20 wk of age; a* = redness at 20 wk of age; b* = yellowness at 20 wk of age; Aswt = upper asymptote (estimated growth curve parameter); tmid = inflection point at 50% asymptote (estimated growth curve parameter); scale = constant that is proportional to the overall growth rate (estimated growth curve parameter). [file 1471-2156-12-14-S1.PDF]

Estimated genetic parameters (heritabilities and correlations with standard errors) for different traits in turkey birds.

Trait	BW17(Kg)	BW40(Kg)	BW60(Kg)	BW80(Kg)	BW120(Kg)	[1]PBM(%)	BrL(mm)	BrW(mm)	PDL(%)	pHu	L*	a*	b*	As_{wt}(Kg)	t_{mid}(day)	[1]scale(day)
**BW17(Kg)**	**0.12(0.20)**	0.97(0.01)	0.91(0.04)	0.86(0.06)	0.88(0.06)	0.16(0.48)	0.54(0.23)	0.87(0.08)	0.13(0.35)	0.03(0.58)	0.58(0.87)	-0.80(0.69)	0.49(0.57)	0.90(0.06)	-0.32(0.40)	0.28(0.34)
**BW40(Kg)**	0.82(0.01)	**0.32(0.22)**	0.96(0.02)	0.91(0.07)	0.91(0.09)	-0.03(0.33)	0.84(0.36)	0.99(0.24)	0.44(0.36)	-0.29(0.37)	0.36(0.38)	-0.18(0.34)	0.68(0.27)	0.98(0.13)	-0.53(0.31)	0.05(0.34)
**BW60(Kg)**	0.74(0.02)	0.82(0.01)	**0.39(0.26)**	0.99(0.007)	0.96(0.02)	-0.02(0.29)	0.90(0.23)	0.89(0.16)	0.67(0.32)	-0.29(0.33)	0.46(0.41)	-0.07(0.29)	0.74(0.21)	0.93(0.09)	-0.63(0.27)	-0.34(0.30)
**BW80(Kg)**	0.68(0.02)	0.79(0.02)	0.90(0.009)	**0.42(0.12)**	0.98(0.01)	-0.08(0.27)	0.82(0.11)	0.84(0.10)	0.62(0.21)	-0.32(0.30)	0.34(0.25)	-0.05(0.28)	0.73(0.15)	0.95(0.04)	-0.59(0.27)	-0.42(0.27)
**BW120(Kg)**	0.63(0.03)	0.71(0.02)	0.82(0.01)	0.86(0.01)	**0.40(0.12)**	-0.09(0.27)	0.91(0.26)	0.96(0.15)	0.49(0.25)	-0.45(0.28)	0.70(0.45)	-0.18(0.26)	0.71(0.23)	0.98(0.01)	-0.46(0.34)	-0.46(0.26)
**PBM (%)**	0.25(0.06)	0.23(0.06)	0.26(0.06)	0.21(0.06)	0.27(0.06)	**0.30(0.10)**	0.24(0.28)	0.20(0.28)	-0.15(0.30)	0.15(0.31)	-0.02(0.27)	-0.18(0.26)	0.06(0.29)	0.02(0.28)	0.34(0.35)	0.27(0.30)
**BrL(mm)**	0.20(0.05)	0.21(0.04)	0.29(0.04)	0.30(0.04)	0.35(0.04)	0.15(0.04)	**0.15(0.06)**	0.68(0.17)	0.50(0.26)	-0.22(0.33)	0.32(0.27)	0.02(0.29)	0.86(0.14)	0.77(0.14)	-0.14(0.41)	-0.48(0.29)
**BrW(mm)**	0.37(0.04)	0.34(0.04)	0.43(0.04)	0.45(0.03)	0.56(0.15)	0.38(0.03)	0.51(0.02)	**0.17(0.07)**	-0.17(0.31)	0.09(0.33)	0.14(0.28)	-0.33(0.25)	0.42(0.26)	0.90(0.08)	-0.12(0.41)	-0.20(0.34)
**PDL (%)**	0.07(0.05)	0.09(0.05)	0.14(0.04)	0.11(0.04)	0.11(0.04)	0.01(0.04)	0.15(0.03)	0.06(0.03)	**0.12(0.06)**	-0.99(0.09)	0.25(0.29)	0.52(0.23)	0.71(0.21)	0.37(0.28)	-0.83(0.27)	-0.66(0.26)
**pHu**	0.06(0.04)	0.00(0.04)	0.03(0.03)	-0.04(0.04)	0.05(0.05)	0.21(0.04)	-0.04(0.04)	0.11(0.03)	-0.18(0.03)	**0.09(0.04)**	-0.42(0.29)	-0.71(0.21)	-0.71(0.21)	-0.40(0.30)	0.25(0.42)	0.32(0.35)
**L***	0.09(0.05)	0.12(0.05)	0.18(0.05)	0.19(0.05)	0.20(0.05)	0.10(0.05)	0.14(0.04)	0.13(0.04)	0.20(0.03)	-0.02(0.03)	**0.27(0.09)**	-0.45(0.22)	0.66(0.18)	0.52(0.21)	0.31(0.30)	0.01(0.33)
**a***	-0.07(0.05)	-0.04(0.05)	-0.05(0.06)	-0.08(0.06)	-0.07(0.05)	0.05(0.05)	-0.07(0.04)	-0.03(0.04)	0.24(0.03)	-0.04(0.03)	-0.35(0.03)	**0.30(0.09)**	0.05(0.29)	-0.26(0.25)	-0.46(0.34)	-0.30(0.30)
**b***	-0.01(0.05)	0.09(0.05)	0.10(0.05)	0.13(0.05)	0.15(0.05)	0.05(0.04)	0.08(0.04)	0.08(0.03)	0.17(0.03)	-0.10(0.03)	0.41(0.03)	0.09(0.04)	**0.15(0.05)**	0.64(0.19)	-0.33(0.37)	-0.48(0.29)
**As_{wt}(Kg)**	0.59(0.04)	0.64(0.03)	0.66(0.03)	0.67(0.02)	0.92(0.007)	0.32(0.05)	0.28(0.03)	0.50(0.03)	0.09(0.04)	0.13(0.04)	0.16(0.05)	-0.04(0.05)	0.12(0.04)	**0.30(0.10)**	-0.27(0.39)	-0.30(0.31)
**t_{mid}(day)**	-0.13(0.04)	-0.16(0.04)	-0.30(0.03)	-0.42(0.03)	0.08(0.04)	0.01(0.04)	-0.01(0.04)	0.03(0.03)	0.002(0.04)	0.17(0.04)	-0.05(0.04)	0.07(0.04)	0.02(0.04)	0.43(0.03)	**0.05(0.04)**	0.64(0.26)
**scale(day)**	0.20(0.05)	0.18(0.05)	-0.05(0.04)	-0.36(0.04)	0.02(0.04)	0.18(0.04)	-0.04(0.04)	0.01(0.04)	0.03(0.04)	0.20(0.04)	-0.06(0.04)	0.10(0.05)	-0.04(0.04)	0.34(0.04)	0.80(0.01)	**0.11(0.06)**

Heritabilities (diagonal), genetic correlations (above the diagonal) and phenotypic correlations (below the diagonal) are presented with standard errors (in parenthesis) for the different traits.

BW01, BW17, BW40, BW60, BW80, and BW120 are the BW at days 1,17, 40, 60, 80, and 120 of age; PBM = percentage breast meat at 20 wk of age; BrL = breast length at 20 wk of age; BrW = breast width at 20 wk of age; PDL = percent drip loss at 20 wk of age; pHu = ultimate pH at 20 wk of age; L* = lightness at 20 wk of age; a* = redness at 20 wk of age; b* = yellowness at 20 wk of age; As_{wt} = upper asymptote (estimated growth curve parameter); t_{mid} = inflection point at 50% asymptote (estimated growth curve parameter); scale = constant that is proportional to the overall growth rate (estimated growth curve parameter).

[1] The genetic correlations for PBM and scale had high standard errors of estimates.
